# Beyond Automation: A Conceptual Framework for Medical Responsibility and Human Competencies in AI-Driven Healthcare

**DOI:** 10.3390/healthcare14213650

**Published:** 2026-10-28

**Authors:** Vittoradolfo Tambone, Laura Leondina Campanozzi, Gianmarco Di Palma, Flavia Giacomobono, Pietro Refolo, Antonio Oliva, Francesco De Micco

**Affiliations:** 1 Research Unit of Bioethics and Humanities, Campus Bio-Medico University of Rome, 00128 Rome, Italy; v.tambone@unicampus.it (V.T.); g.dipalma@policlinicocampus.it (G.D.P.); f.demicco@policlinicocampus.it (F.D.M.); 2 Department of Clinical Affairs, Campus Bio-Medico University Hospital Foundation, 00128 Rome, Italy; flavia.giacomobono@gmail.com; 3 Section of Bioethics and Medical Humanities, Department of Health Care Surveillance and Bioethics, Università Cattolica del Sacro Cuore, 00168 Rome, Italy; pietro.refolo@unicatt.it; 4 Section of Legal Medicine, Department of Health Surveillance and Bioethics, Fondazione Policlinico A. Gemelli IRCCS, Università Cattolica del Sacro Cuore, 00168 Rome, Italy; antonio.oliva@unicatt.it

**Keywords:** artificial intelligence, medical ethics, clinical reasoning, responsibility, agentic AI, embodied AI, human oversight, informed consent, professional exigibility, personalism, patient–physician relationship, healthcare governance

## Abstract

**Highlights:**

**What are the main findings?**

- AI-driven medicine requires human governance competencies based on four irreducible questions (Truth, Good, Person, and Context) and epistemic independence, rather than searching for residual human tasks.
- We propose “professional exigibility” as a jurisdiction-sensitive analytical medico-legal category for practice.

**What are the implications of the main findings?**

- Meaningful human oversight of medical AI demands genuine epistemic competence and independence, moving beyond the mere formal presence of a human in the decision loop.
- Professional exigibility provides an analytical approach to assessing when validated AI capabilities may become reasonably expected in standard professional practice.

**Abstract:**

**Background/Objectives:** Artificial intelligence (AI) is increasingly embedded in clinical reasoning, documentation, prediction, communication, and delegated digital and physical action via agentic and embodied systems. The central ethical challenge is no longer limited to identifying which tasks AI can automate, but determining which human competencies become essential as technological capabilities expand. **Methods:** This concept paper employs a five-step analysis: technological horizon mapping, selective synthesis of work-transformation evidence, conceptual analysis of medical action, normative synthesis, and medico-legal interpretation. **Results:** We propose a four-domain framework addressing four core questions of responsible medical action: epistemic (What is true?), normative (What ought to be done?), anthropological–relational (Who is the subject of care?), and systemic–hermeneutic (What does this mean here and now?). Grounded in a conception of technology as augmentative alteration, the framework recognises that technological capabilities can enhance beneficial action but also amplify harm; technological capability alone therefore cannot determine its appropriate use. We also distinguish interpersonal relationships from clinical human–machine interaction, propose professional exigibility as an analytical medico-legal category, and argue that meaningful human oversight requires more than mere human presence. **Conclusions:** The findings outline key implications for clinical practice, medical education, healthcare organizations, regulation, and a future empirical research agenda focused on human–AI error convergence.

## 1. Introduction

The development of contemporary AI is occurring at a speed that makes any stable description provisional. The conceptual work underlying this paper had to broaden repeatedly: from generative AI to agentic AI, from predominantly cognitive systems to embodied and physical AI, and then to the possible future implications of quantum-enabled computation. These are not successive replacements of one paradigm by another. They are enlargements of the object of analysis and can coexist within the same clinical ecosystem. This pace creates a methodological problem for medicine. If medical education, professional regulation, and medico-legal analysis are organised around the capabilities of one generation of tools, they risk becoming obsolete before implementation. More stable categories are therefore needed: categories that remain relevant even as technologies change. The hypothesis advanced here is that increasing technological capability progressively shifts a distinctive component of medical competence from the production of inference toward the responsible governance of inference and action. Existing scholarship has extensively examined the ethical implications of AI in healthcare, including epistemic, normative, and accountability-related challenges [1]. These issues have also been addressed through complementary approaches to responsible technological development and clinical use. Ethical and institutional governance approaches, such as the World Health Organization’s guidance, establish principles concerning human autonomy, safety, transparency, and accountability [2]. These principles are complemented by frameworks such as FUTURE-AI, which provides recommendations for trustworthy development and implementation [3], and by clinical evaluation guidelines such as DECIDE-AI, which address safety, clinical utility, and human factors [4]. Alongside these contributions, research on human–AI collaboration has highlighted the distinction between algorithmic prediction and professional judgment [5]. These approaches provide essential ethical, clinical, and governance perspectives. A related question, however, receives less explicit attention within these selected contributions: as AI increasingly participates in clinical inference and action, what integrated set of human competencies is required to govern technologically mediated medical practice?

Against this background, the originality of the present framework does not lie in introducing previously unknown ethical concepts. Epistemic reliability, normative deliberation, person-centred care, contextual judgment, and professional responsibility all have established traditions. The proposed contribution lies instead in their functional integration within a single governance architecture for AI-mediated medicine.

Three elements define this contribution. First, the framework shifts the analytical focus from identifying tasks that remain exclusively human to identifying the competencies required to govern increasingly distributed inference and action. Second, it organises these competencies around four non-substitutable questions: Truth, Good, Person, and Context. An adequate response to one question cannot be assumed to resolve the others. Third, the framework considers these competencies not merely as safeguards against technological substitution but also as potential enabling conditions for responsible innovation. The framework also connects this governance architecture with professional exigibility, examining when validated technological capabilities become sufficiently established, accessible, and clinically integrated for their consideration to be reasonably expected of professionals. The proposal is therefore not intended as a replacement for existing AI ethics or governance approaches, but as an integrative clinical and medico-legal architecture linking technological capability, evidence, judgment, and responsible action (Table 1).

The central thesis can therefore be stated concisely: the more capable the technology becomes, the less sufficient technical competence alone becomes for governing its use. This does not diminish technical expertise. It increases the importance of the human capacity to judge what is true enough to act upon, what ought to be done, who the patient is as a person, and how general knowledge should be interpreted within a concrete and changing system.

## 2. Methodological Approach

This paper is a theoretical and conceptual analysis rather than a systematic review or technological forecast. Its purpose is to construct a transparent cross-disciplinary framework that can subsequently generate empirical and medico-legal research. Source selection was purposive rather than exhaustive, guided by the contribution of philosophical, clinical, technological, institutional, and medico-legal sources to five analytical objectives: mapping emerging AI capabilities, examining potential changes in professional work, analyzing medical judgment and responsibility, identifying ethical and anthropological requirements, and assessing implications for professional standards and accountability. The analysis was organized in five phases.

**Phase 1—Technological horizon mapping.** Generative, agentic, and embodied AI were mapped as expanding modalities of technological agency. Quantum computing was retained as a possible future computational discontinuity rather than treated as a mature fourth stage.

**Phase 2—Complementary signals of work transformation.** Anthropic, Barclays, and McKinsey were selected deliberately as three different prospective lenses: observed real-world exposure to cognitive AI; scenario analysis concerning physical AI and robotics; and industrial market scenarios. These sources were selected to consider three complementary dimensions of technological change—observed AI exposure, the potential extension of automation to physical tasks, and possible industrial scaling—and to examine their implications for the reorganization of professional activities rather than occupational replacement alone. They are not treated as a comprehensive literature review and are not used to predict medicine directly.

**Phase 3—Conceptual analysis of medical action.** The analysis distinguishes inference from judgment, interaction from interpersonal relation, technological capability from responsibility, standard of care from professional exigibility, and statistical correlation from contextual clinical interpretation. These distinctions were examined to identify clinically and medico-legally relevant differences that would be obscured if the corresponding concepts were treated as equivalent.

**Phase 4—Normative synthesis**. The conceptual distinctions established in Phase 3 were organised according to the questions they raise for responsible medical action. This conceptual synthesis led to the identification of four analytically distinct domains: Truth, Good, Person, and Context. Each addresses a question that cannot be fully answered by the others. These questions are not ontologically homogeneous; they represent distinct conditions of responsible clinical action. The resulting framework was then examined in relation to key challenges of AI-mediated healthcare, including AI-supported inference, automation bias, meaningful human oversight, patient participation, contextual interpretation, and professional responsibility.

**Phase 5—Medico-legal and healthcare implications.** The framework was then applied to human oversight, automation bias, informed consent, standards of professional conduct, distributed responsibility, and the jurisdiction-dependent question of what can reasonably be demanded from professionals and healthcare systems.

The explicit description of this analytical procedure allows the conceptual derivation of the framework to be critically examined, including the relevance of the distinctions identified, their organisation into four domains, and the possibility of alternative conceptual configurations. This transparency supports the critical assessment of the proposed framework without implying the procedural reproducibility of a systematic literature review.

## 3. Expanding Modalities of Technological Agency

### 3.1. Generative, Agentic, and Embodied AI

Generative AI produces text, images, code, multimodal summaries, and probabilistic proposals. Agentic AI adds delegated digital action: systems can plan, call tools, coordinate multi-step workflows, retrieve information, and execute tasks with varying degrees of autonomy. Embodied AI adds a further dimension: action within the physical environment through robotics, sensors, computer vision, and autonomous or semi-autonomous devices.

**Figure 1:**
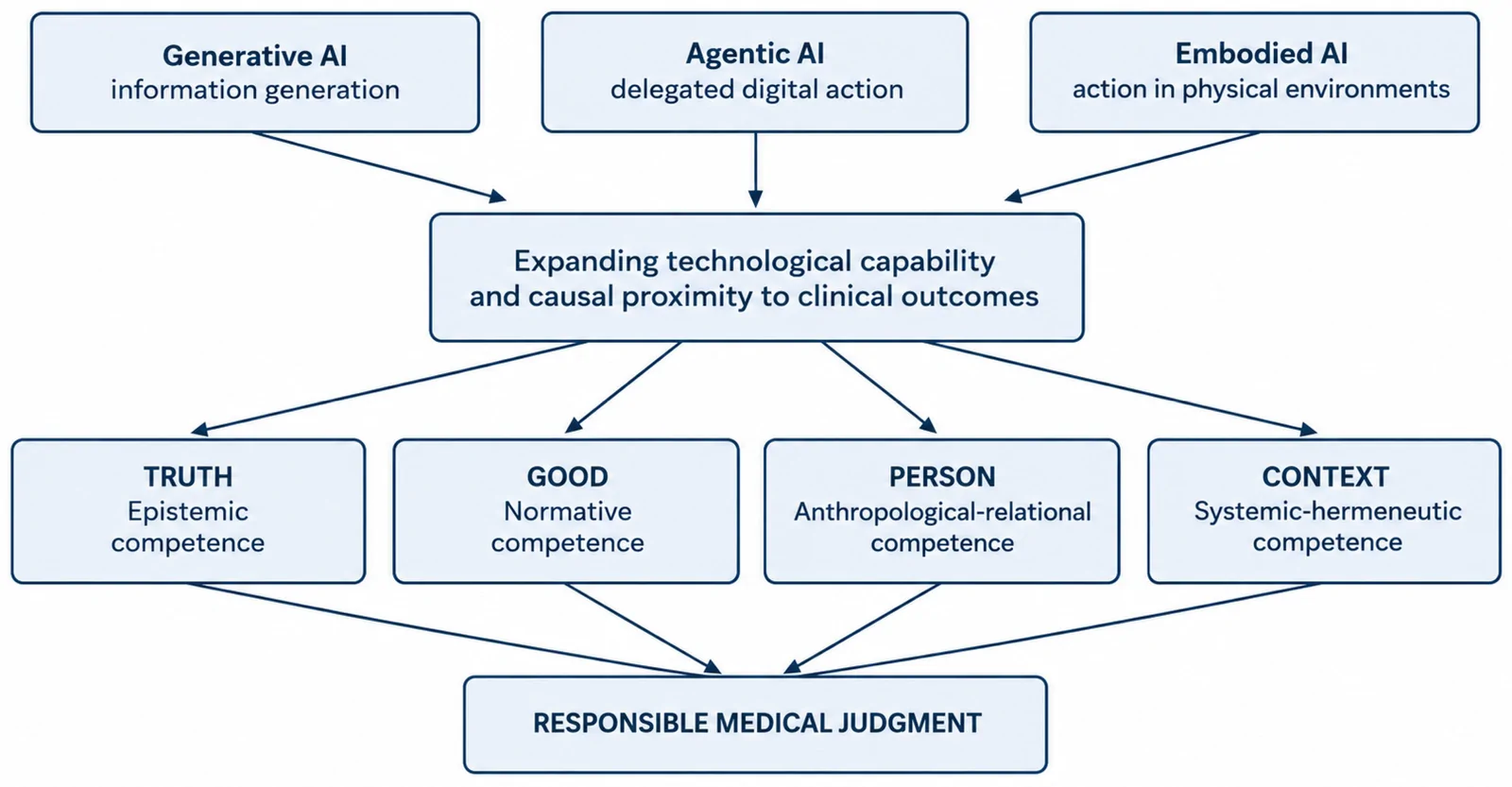
Expanding technological capability and the four governance competencies proposed for AI-driven medicine.

These categories are neither mutually exclusive nor proposed as a deterministic technological sequence. They identify progressively broader modalities through which artificial systems may participate in human action. In healthcare, the distinction matters because movement from information production to delegated action increases causal proximity between AI output and patient outcomes, thereby increasing the demands placed on governance and responsibility (Figure 1).

### 3.2. Quantum Computing as a Possible Future Discontinuity

Quantum computing should not be regarded as a fourth mature stage of medical AI. Current evidence does not demonstrate a consistent quantum advantage across healthcare applications, and its future clinical relevance remains uncertain [6]. Nevertheless, emerging research is exploring its potential in biomedical applications, including biomarker discovery and radiotherapy planning [7,8,9]. Its relevance to the present analysis is therefore conceptual rather than predictive: it illustrates how future computational architectures may evolve beyond current AI technologies.

Such developments could expand the speed, scale, and complexity of prediction, simulation, and decision support, without necessarily improving the reliability of clinical information or the appropriateness of medical decisions. Greater computational capability would still require human judgment concerning the validity of technological outputs, the goods pursued through clinical action, the recognition of the patient as a person, and the interpretation of evidence within the concrete context of care.

Quantum computing thus serves as an illustrative case of possible technological discontinuity, reinforcing the central argument that the fundamental questions of Truth, Good, Person, and Context remain relevant to responsible medical judgment regardless of changes in computational architecture.

## 4. Technology as Augmentative Alteration and AI as a Domain Activator

### 4.1. Augmentative Alteration

The framework adopts a conception of technology as an augmentative alteration of human action. Genealogy is deliberately dialogical rather than reductive. Aristotle distinguishes technē from practical wisdom; Aquinas distinguishes art from prudence while integrating both within human action; contemporary authors such as Spaemann, Agazzi, and MacIntyre provide additional resources for evaluating when technological mediation strengthens human practice and when it risks displacing the agent or distorting the ends of an activity [10,11,12,13,14].

By augmentative alteration we mean that technology changes the effective possibilities of action while remaining ordered, when appropriately governed, to the end of the human practice it serves. The category is therefore not synonymous with transhumanist substitution. It does not presume that every limitation is a defect to be technically eliminated, nor that technological expansion is equivalent to human improvement.

**Figure 2:**
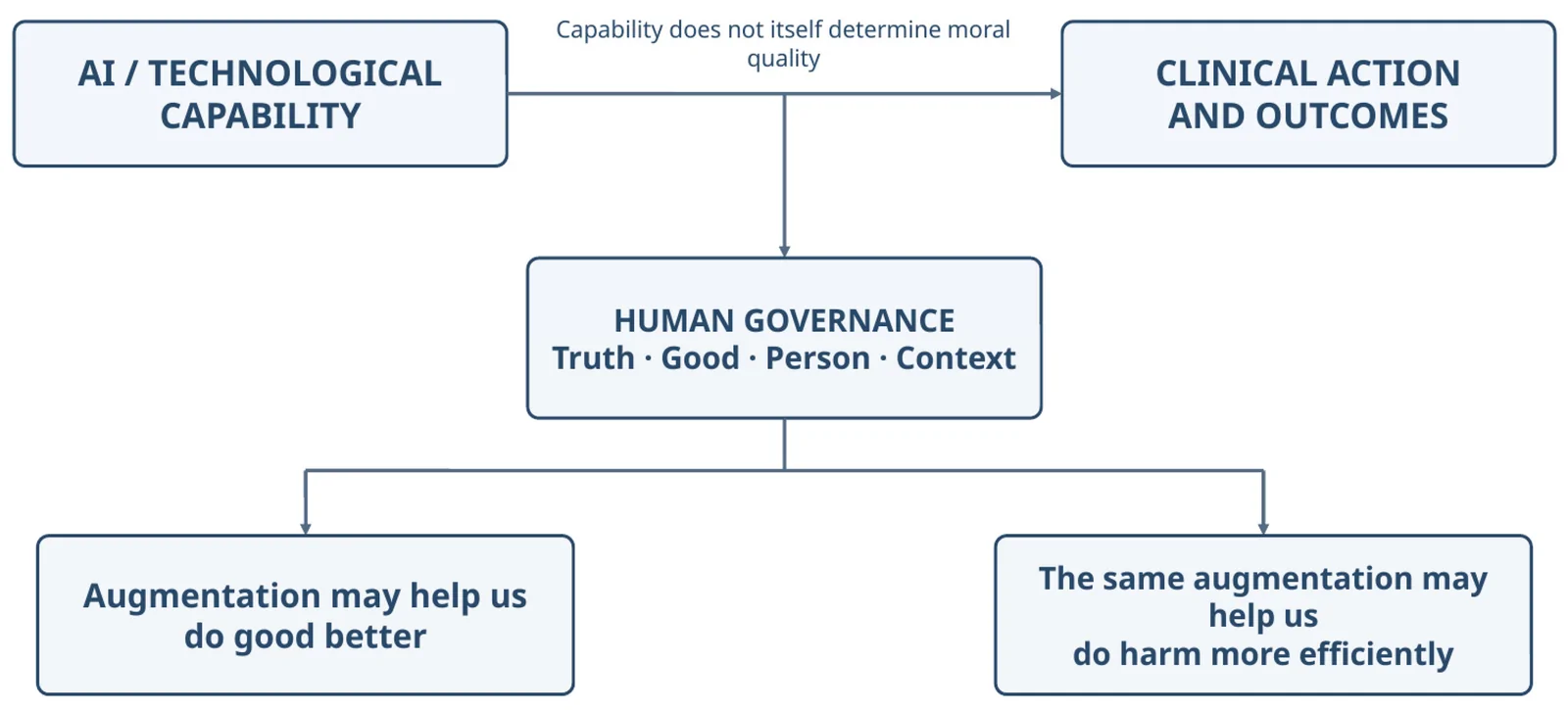
Technological augmentation increases capability but does not determine the moral quality of its use.

Most importantly, augmentation is normatively indeterminate. The same increase in technical capacity can help a professional do good better or do harm more efficiently. Increased capability is therefore not equivalent to increased goodness. This asymmetry explains why growing technological power increases rather than eliminates the need for normative competence (Figure 2).

### 4.2. AI as a Domain Activator

AI is often discussed as though it were one more specialised domain. This is misleading. Kissinger, Schmidt, and Huttenlocher describe AI as a transformative technology whose effects cross multiple domains of knowledge and action; the Italian edition explicitly uses the expression “attivatore di domini” (domain activator) early in the text [15,16]. We retain that expression because it captures an important structural point: AI does not merely add a new specialty to healthcare but activates and transforms pre-existing domains by being vertically instantiated within them.

The resulting architecture is: general-purpose capability → vertical domain instantiation → systemic integration → professional governance. Medical AI must therefore be clinically vertical—radiology, pathology, legal medicine, surgery, primary care, pharmacy, rehabilitation—and simultaneously systemic, because outputs from one domain alter decisions and constraints in others. This dual requirement makes domain expertise and systems thinking complementary rather than competing forms of competence.

## 5. Complementary Signals of Work Transformation

The following sources are intentionally heterogeneous. They are used to illuminate different dimensions of technological transformation, not to provide a single forecast and not to infer medical outcomes directly.

### 5.1. Anthropic: Observed Exposure

Anthropic’s 2026 report introduced Observed Exposure, combining theoretical LLM capability with real-world usage and weighting automated and work-related uses more heavily. Actual use remained substantially below theoretical capability; no systematic increase in unemployment was identified in highly exposed occupations, although there was suggestive evidence of slower entry by younger workers [17]. The important lesson is that capability does not translate automatically into occupational substitution.

### 5.2. Barclays: From Cognitive to Physical AI

Barclays’ scenario work broadens the analysis from cognitive automation toward physical AI and robotics. Its value here is not evidentiary equivalence with labour-market data, but the recognition that embodiment expands automation into tasks previously protected by sensorimotor and physical constraints [18].

### 5.3. McKinsey: Industrial Scaling

McKinsey’s scenarios estimate that the global robotics industry, approximately USD 21 billion in 2022, could reach roughly USD 190–910 billion by 2040. This is a hypothetical range rather than a prediction and concerns the robotics industry overall, not healthcare robotics specifically [19].

### 5.4. Interpretation

Taken together, these signals support only a cautious proposition: before occupations disappear, the internal distribution of tasks, coordination, verification, and responsibility may change substantially. Although these sources do not establish whether or to what extent such changes will occur in healthcare, they provide a broader perspective from which to examine their possible implications for medical practice. In medicine, the relevant question is therefore not whether a report predicts the disappearance of physicians, but whether and how technologically mediated task recomposition could affect the structure of professional judgment.

## 6. From Innovation to Governance: Truth and the Good

Peter Thiel’s contrarian question asks what important truth very few people agree with the respondent about [20]. The heuristic is influential because radical innovation often begins when someone recognises something that others do not see. We retain this reference because it illuminates a real source of innovation: the capacity to challenge consensus.

At the same time, the argument uses concepts that remain philosophically underdetermined within the entrepreneurial framework itself. Truth performs a central epistemic function without an explicit theory of truth, while innovation and progress are positively valued without an explicit account of the good by which progress should be assessed. This is Thiel’s unresolved problem, not a criticism that an entrepreneurship book ought to become a metaphysical treatise. The limitation becomes decisive when the heuristic is transferred into medicine, where novelty is not identical to truth and innovation is not identical to good care.

The governance of AI-driven medicine therefore requires four questions that technical performance alone cannot answer. They are not four homogeneous metaphysical categories. They are four irreducible conditions of responsible medical action: What is true? What ought to be done? Who is the subject of care? What does this mean here and now? These four questions and the corresponding governance competencies are summarised in Table 2.

## 7. The Four-Domain Human Governance Framework

The four domains are proposed as analytically irreducible and jointly constitutive dimensions of responsible medical judgment. Each addresses a necessary question that cannot be adequately resolved by the others: the reliability of knowledge (Truth), the ends and justification of action (Good), the recognition of the patient as a person and participant in care (Person), and the interpretation of evidence within specific clinical and organisational circumstances (Context). Their irreducibility does not imply independence, as responsible judgment requires their integration. The framework does not claim to provide an exhaustive taxonomy of professional competencies, but rather a structured account of the fundamental questions that guide responsible medical action. Capacities such as practical wisdom and critical–reflective thinking, as well as collaborative forms of interprofessional judgment, contribute to addressing and integrating these questions rather than constituting additional domains of the same analytical order.

*From a Conceptual Framework to a Cross-Cutting Governance Layer*

The additional analytical contribution of the proposed framework lies not merely in organising four established dimensions of medical responsibility, but also in their potential translation into the conceptual and operational design of AI systems used in healthcare. Truth, Good, Person, and Context may be conceived as constitutive dimensions of a cross-cutting governance layer applicable across heterogeneous clinical AI systems, including generative, predictive, agentic, and embodied AI. Rather than functioning solely as external ethical constraints or retrospective assessment criteria, these dimensions could inform the design of AI-mediated clinical workflows, shaping how systems formulate recommendations, communicate uncertainty, identify normative trade-offs, support patient participation, and interpret information within specific clinical contexts.

Such a layer would neither impose predetermined moral conclusions nor replace professional deliberation with algorithmic judgment. Its purpose would be to support the clinician’s critical–reflective reasoning and epistemic independence during the execution of clinical tasks by making relevant assumptions, alternatives, limitations, and decision criteria available for critical evaluation. The framework thus suggests a possible transition from principles of human governance towards an operationally testable approach to human–AI collaboration. Its distinctive analytical contribution lies in identifying the conditions under which technological outputs may be integrated into responsible medical action, while providing a structured basis for the future design, implementation, and empirical evaluation of governance mechanisms across different AI systems. This proposed architectural application remains a research hypothesis rather than an implemented or validated technological solution.

### 7.1. Truth: Epistemic Competence

Truth is not included as a decorative philosophical category. It is a clinical condition of informed consent and professional responsibility. A patient cannot meaningfully consent to a decision if the information offered is materially false, misleading, or presented with unjustified certainty. Likewise, professional accountability presupposes a responsible relation to evidence.

In AI-driven medicine, epistemic competence concerns the entire clinical pathway: interpretation of symptoms and diagnostic tests; appraisal of clinical-trial evidence; use of epidemiological priors; evaluation of model performance and distribution shift; therapeutic prediction; and follow-up as a reality constraint capable of revising the original framing. Generative AI introduces additional epistemic risks, including plausible but inaccurate or fabricated clinical information, which further underscores the need to critically assess the reliability of technological outputs [21]. The relevant question is not whether the system generates a probability, but whether that probability can reasonably be treated as knowledge for this decision, in this patient, under these conditions.

### 7.2. Good: Normative Competence and Professional Excellence

The question of the good arises first within the ethics of well-executed work: good medical practice implies professional excellence, not merely technical completion of a task. The language of “best practice” already contains a normative claim. Best according to which good?

An algorithm can optimise a defined objective function, but the choice of that objective is normatively prior to optimisation. Survival, quality of life, autonomy, symptom relief, equity, proportionality, patient preference, and stewardship can converge, but they can also conflict. Normative competence is the capacity to deliberate among these goods while remaining oriented toward professional excellence and the integral good of the patient within the common good. Optimization therefore presupposes, rather than produces, an account of what should count as good.

### 7.3. Person: Anthropological–Relational Competence

The international language of humanisation, person-centred care, rights, autonomy, and patient–physician relationship presupposes an anthropology, whether acknowledged or not. Claims about “relationship” between a person and a robot or AI should therefore define what philosophical concept of relation is being used.

This paper explicitly rejects the fiction of metaphysical neutrality. Its anthropological reference is the personalist tradition of the Lublin School, placing Karol Wojtyła and Mieczysław A. Krąpiec in dialogue without treating them as interchangeable. This is analogous to Wojtyła’s own capacity to place Thomistic metaphysics and Schelerian phenomenology in dialogue without collapsing one into the other [22,23]. Wojtyła’s analyses of action, self-determination, participation, and responsibility and Krąpiec’s metaphysical realism provide different but dialogical resources for understanding the person as more than an emergent function of a system. More specifically, Wojtyła’s emphasis on action and participation helps elucidate the patient’s role as an active participant in care, while Krąpiec’s metaphysical realism provides an ontological foundation for understanding personhood as irreducible to observable functions or technological performance.

Within this framework, an AI or robot can produce effective interaction: an exchange of actions that may influence behaviour, emotion, trust, adherence, or even clinical outcomes. Some effects on the patient may resemble effects experienced in a human relationship. The difference is that these effects do not signify the intentional attitude, concern, commitment, or responsibility of another human subject toward the patient. Functional or phenomenological similarity therefore does not establish ontological equivalence. We consequently reserve interpersonal relationship for person-to-person relation and use interaction for clinically meaningful exchange with an artificial system.

This distinction does not diminish the possible clinical value of AI interaction. It clarifies responsibility. A system may generate an empathic-seeming response without itself becoming the bearer of professional responsibility or reciprocal personal commitment. This is particularly relevant where healthcare policies call for humanisation while digital mediation increases.

### 7.4. Participation and the Democratisation of AI in Medicine

Democratisation cannot be reduced to making AI tools technically available to more people. In personalist language, participation means taking part. A democratised AI ecosystem should therefore increase the ability of patients and professionals to take part consciously in understanding, deliberation, and decision-making rather than converting them into passive recipients of algorithmically optimised pathways.

This interpretation is compatible with international commitments to human rights, dignity, informed consent, and people-centred healthcare, including the Oviedo Convention, UNESCO’s Universal Declaration on Bioethics and Human Rights, and the WHO framework on integrated people-centred health services [24,25,26].

### 7.5. Context: Systemic–Hermeneutic Competence

Context is not a residual category containing everything that does not fit elsewhere. It has two components. The systemic component concerns relationships among variables, organisations, resources, epidemiology, workflows, feedback loops, and non-linear effects. The hermeneutic component concerns meaning: what the available correlations and evidence mean for this particular patient in this particular history.

AI can personalise outputs at extraordinary scale. Personalisation, however, is not identical to contextual understanding. Contextual competence also requires recognising that clinically relevant dimensions of the patient’s condition and care needs may be absent or underrepresented in the available data, limiting how fully algorithmic outputs can reflect the complexity of the individual clinical situation [27]. Correlation becomes clinically meaningful only through interpretation within a system and a history. Contextual competence therefore converts general evidence into situated judgment while remaining open to correction through follow-up.

### 7.6. Clinical Illustration: AI-Supported Assessment of Adverse Drug Events in Pediatric Congenital Heart Disease

The analytical contribution of the proposed framework can be illustrated through a hypothetical clinical scenario involving adverse drug events (ADEs) in children with congenital heart disease (CHD). These patients may require complex pharmacological regimens, while age-related physiological differences, polypharmacy, and comorbidities complicate the assessment of drug-related risks [28,29]. Recent research has explored machine learning approaches to identifying ADEs in this population, providing a relevant example of how algorithmic prediction might contribute to clinical decision-making [30].

Consider a child with complex congenital heart disease receiving multiple medications. An AI-based clinical decision-support system identifies an elevated probability of an adverse drug event associated with one component of the treatment regimen. Based on its predictive model, the system suggests reassessing the treatment and identifies modification of the suspected medication as a possible risk-reduction strategy.

A decision-making process focused exclusively on predictive accuracy might prioritise reducing the estimated probability of the adverse event. However, even if the prediction is statistically reliable, its clinical implications remain underdetermined. The proposed four-domain framework makes this underdetermination explicit and provides a structured approach to examining the considerations relevant to clinical action.

Truth—Epistemic competence. The clinician must first assess whether the predicted risk is supported by sufficiently reliable evidence, whether the model has been validated in a clinically comparable pediatric population, and whether its input variables adequately represent the child’s condition. The system’s probability estimate must not be confused with a confirmed adverse drug event or with evidence that modifying treatment will necessarily improve outcomes. Relevant uncertainties, alternative explanations, and potential limitations of the underlying data must therefore be made explicit.

Good—Normative competence. Even when the predicted risk is reliable, reducing that risk is not necessarily equivalent to providing better care. Modifying the medication might reduce the probability of an adverse event while increasing the risk of deterioration of the underlying cardiac condition. An appropriate decision therefore requires consideration of expected benefits, potential harms, therapeutic alternatives, proportionality, and the patient’s overall clinical interests. Optimising a single predictive endpoint cannot, by itself, determine the appropriate therapeutic objective.

Person—Anthropological–relational competence. The patient cannot be reduced to the biological variables represented in the model. Clinical deliberation must involve communication with the child’s parents or legal guardians and, according to the child’s age and maturity, appropriate participation by the child. Previous treatment experiences, concerns, preferences, and the practical consequences of alternative therapeutic strategies may also be relevant to shared decision-making. Algorithmic recommendations can inform this process but cannot replace the responsibilities inherent in interpersonal clinical care.

Context—Systemic–hermeneutic competence. The clinical significance of the prediction depends on the child’s actual condition, the severity of the cardiac disease, concomitant treatments, the availability of alternative medications, the feasibility of close monitoring, and the organisational resources of the healthcare setting. Clinically relevant circumstances may also be absent or inadequately represented in the data used by the algorithm. The same predictive output may therefore support different reasonable decisions in different clinical contexts.

Integration and potential decision change. Consider two possible scenarios. In the first, the predicted adverse-event risk is supported by reliable evidence, an equally effective therapeutic alternative is available, and the child’s clinical circumstances allow treatment to be modified safely. In the second, the suspected medication is essential to maintaining clinical stability, the available alternatives carry substantial risks, and close monitoring is feasible. In the latter situation, continuing treatment under an appropriately adjusted monitoring plan may be more reasonable than the modification initially suggested by the AI system. These scenarios are illustrative possibilities rather than treatment recommendations for an actual patient.

The additional analytical value of the four-domain framework lies in making explicit why the same algorithmic prediction does not necessarily justify the same clinical action. Truth concerns the epistemic status of the prediction; Good addresses the ends and proportionality of intervention; Person ensures recognition of the patient as a participant in care; and Context concerns the interpretation of evidence within specific clinical and organisational circumstances. These dimensions interact rather than operate as independent, sequential checks.

The same scenario also illustrates the potential function of a cross-cutting AI governance layer. Rather than merely presenting a ranked recommendation, an AI system designed to incorporate the four-domain framework could prompt the clinician to examine the validity of the prediction, compare therapeutic objectives, consider patient participation, and identify contextual information that might alter the decision. Such a system would not automatically determine the morally or clinically appropriate course of action. Instead, it could support clinical decision-making while encouraging the clinician’s critical–reflective reasoning and capacity to question the initial recommendation.

This illustration demonstrates the conceptual applicability of the framework and identifies a possible direction for computational implementation. It does not establish empirical superiority over existing decision-support systems or governance frameworks. Any such comparative advantage would require prospective evaluation of clinical decision quality, error detection, automation bias, clinician independence, patient participation, and relevant health outcomes.

## 8. Medical Responsibility Under Technological Change

### 8.1. Standard of Care and Professional Exigibility

Standards of care are context-sensitive and evolve with evidence, professional practice, technological availability, and organisational conditions [31,32,33]. The medico-legal problem created by AI is therefore symmetrical. Premature or uncritical delegation to an opaque system may fall below reasonable professional conduct. Conversely, non-use of AI should not become professionally relevant merely because a system is technically available or has shown promising performance. Relevance arises only when the technology has reached a sufficient level of scientific validation, clinical appropriateness, accessibility, integration into professional practice, and organisational support to make its consideration reasonably expected in the concrete case.

Recent medico-legal scholarship has similarly examined how AI capabilities may reshape professional standards without automatically generating duties to adopt or rely on technological systems. In particular, it has emphasised the relevance of institutional validation, transparency, and the conditions under which clinicians can exercise informed judgment [34,35].

Within this framework, professional exigibility is not proposed as a freestanding transnational doctrine or an independent legal duty, or as a replacement for established standards of care. It is used as an analytical medico-legal category that makes explicit a familiar question: what specific conduct could reasonably be demanded of a competent professional in the circumstances actually existing at the time? Unlike professional eligibility, which concerns qualification or suitability to perform a professional role or activity, professional exigibility concerns the reasonable demandability of a specific course of professional conduct. Although context-sensitive standards of care may already incorporate considerations of feasibility and professional circumstances, the analytical contribution of professional exigibility lies in isolating the assessment of whether evolving technological capabilities and professional expectations translate into conduct concretely required of an individual clinician. This distinction is particularly relevant to AI-mediated care, where the ability to use, interpret, question, or override technological outputs depends on both individual competence and conditions established by healthcare organisations and technology providers.

The assessment depends on scientific knowledge, validated technological availability, professional guidance, organisational conditions, the professional’s role and competence, and the concrete circumstances of care. Its legal content must therefore be specified within national medico-legal systems. The Italian Gelli-Bianco framework (Law No. 24/2017, governing patient safety and healthcare professional liability) provides one jurisdiction-specific example of the interaction between professional responsibility, guidelines, good clinical practice, and case-specific professional conduct [36].

Analytically, four questions should remain distinct: first, what conduct or technological consideration formed part of the relevant professional standard; second, whether that conduct was concretely exigible from the professional in the circumstances; third, whether the conduct actually adopted represented a legally relevant departure from what was required; and fourth, whether any such departure had the causal significance required by the applicable legal system. Professional exigibility therefore does not by itself establish liability. It operates as an intermediate inquiry within the assessment of professional conduct, distinguishing the identification of the relevant professional standard from the evaluation of what could reasonably be required in the circumstances, without establishing an independent basis for legal liability.

For AI-mediated care, exigibility should be assessed through a convergent set of factors rather than a single threshold. Relevant factors include: the degree of scientific and external validation; applicability to the relevant patient population and clinical task; regulatory status where applicable; incorporation into guidelines, recommendations, or established good practice; actual availability within the healthcare organisation; the professional’s training and access to adequate information; the possibility of understanding, questioning, and, where appropriate, overriding the output; the availability of organisational support and escalation pathways; and the balance between expected benefit, risk, and reasonable alternatives. No single factor is necessarily decisive.

This assessment is also relational: what can reasonably be demanded of an individual professional depends partly on the conditions created by the healthcare organisation. A requirement to supervise, verify, or override an AI system cannot be treated as meaningful if the professional lacks the time, information, training, authority, or technical and organisational support necessary to perform those functions. Professional and organisational responsibility should therefore be analysed together, without collapsing one into the other.

Consider, for example, two clinicians working in different healthcare organisations, both facing a clinical situation in which the same validated AI decision-support system could provide relevant diagnostic information. In the first setting, the system is integrated into clinical workflows, and the clinician has received appropriate training and has access to the information and support needed to evaluate its outputs. In the second, the system is nominally available, but adequate training and organisational support for its use and oversight are lacking. Although the technological capability is identical, what can reasonably be required of each clinician may differ. Professional exigibility makes this distinction explicit by focusing on the conditions under which technological capabilities translate into concrete professional expectations, without presuming that organisational limitations automatically exclude professional obligations or determine legal liability.

Professional exigibility in this sense should also be distinguished from the justiciability or legal enforceability of healthcare entitlements. The latter asks what a patient can demand from the state, an insurer, or a healthcare institution. The former asks what can reasonably be demanded from the professional. The two are related but not identical. Their comparison nevertheless demonstrates a common point: healthcare obligations are embedded in concrete legal orders rather than floating as jurisdiction-free abstractions.

### 8.2. A Comparative Legal Illustration of Healthcare Exigibility

Italy constitutionally protects health under Article 32. Constitutional Court jurisprudence has described a constitutionally protected irreducible core while also recognising that positive healthcare entitlements are shaped through reasonable balancing and available organisational and financial resources [37,38,39].

In England, the NHS framework creates statutory duties to provide services to meet reasonable requirements, while judicial review traditionally leaves substantial space for resource-allocation decisions; R v Cambridge Health Authority, ex parte B remains a classic illustration of judicial restraint in reviewing treatment and funding decisions [40,41].

In the United States, there is no general federal constitutional entitlement to universal healthcare comparable to Article 32 of the Italian Constitution. Legal entitlement is fragmented across statutes, programmes, insurance arrangements, and contractual structures. EMTALA creates a specific federal obligation concerning emergency screening and stabilisation for individuals presenting to covered hospital emergency departments [42].

South Africa provides a distinct constitutional model. Section 27 of the South African Constitution recognises a right of access to healthcare services while requiring reasonable measures within available resources to achieve progressive realisation, and it separately provides that no one may be refused emergency medical treatment [43].

These systems differ profoundly, which is precisely the point: any application of professional exigibility to AI must be articulated inside the legal architecture governing professional duties, patient entitlements, resources, institutional responsibility, and access in the relevant jurisdiction. Jurisdictional sensitivity should therefore qualify the legal consequences of the concept, not obscure the analytical distinction between what technology can do and what a professional could reasonably be required to do in a concrete setting.

### 8.3. Automation Bias and Meaningful Human Oversight

Human presence does not automatically constitute effective oversight. Automation bias can transform the clinician from an independent evaluator into a procedural confirmer of machine output. Recent scholarship has analysed both the behavioural mechanisms of automation bias and the legal difficulty of attempting to de-bias human oversight through regulation [44,45].

This has a direct implication for the EU AI Act. Article 14 requires high-risk AI systems to be designed and developed so that they can be effectively overseen by natural persons, with oversight measures proportionate to risk, level of autonomy, and context of use. The same provision contemplates the ability to understand system capabilities and limitations, remain alert to automation bias, interpret outputs, and, where appropriate, disregard, override, reverse, or interrupt them. Article 26 places complementary obligations on deployers, including assigning human oversight to persons with the necessary competence, training, authority, and support [46]. Meaningful human oversight therefore requires more than formal human presence: it requires epistemic competence together with practical conditions that make independent judgment possible. A clinician cannot meaningfully supervise a system if he or she lacks sufficient knowledge, authority, time, information, or independence to challenge its output.

Epistemic independence does not require clinicians to reconstruct the internal operations of complex AI systems, but to retain the capacity to critically assess their outputs against clinical evidence, patient-specific information, and professional judgment. Mitigating automation bias therefore requires not only appropriate training but also organisational and procedural safeguards, such as opportunities for independent clinical assessment, access to relevant information about system limitations, and clear procedures for questioning or overriding AI recommendations. These measures should be proportionate to the system’s risks and the clinical context, without imposing verification duties that exceed what can reasonably be expected of individual professionals.

### 8.4. Learned Intermediary, Product Liability, and Distributed Accountability

The learned intermediary doctrine, developed principally in United States pharmaceutical and medical-device law, offers a useful analogy rather than a directly transferable legal solution across jurisdictions. In its traditional form, the physician mediates between manufacturer information and patient care [47]. With complex AI, the analogy becomes strained when system behaviour cannot realistically be reconstructed or independently verified by the individual clinician, or when the conditions for meaningful oversight depend on organisational and technical arrangements beyond the clinician’s control.

European product-liability law is also evolving to address software and digital products, including AI systems within the modernised product-liability framework, while scholarship on autonomous AI increasingly proposes layered or tiered models of responsibility [48,49,50]. At the time of writing, this remains an evolving European framework whose practical operation also depends on national implementation. These developments should not be conflated with professional negligence: product defect, organisational failure, and professional fault may arise from different legal bases and must be assessed separately. The resulting principle should be simple: responsibility can be distributed without becoming dissolved, but distributed causation does not entail undifferentiated liability. Clinicians, healthcare organisations or deployers, developers, and manufacturers may occupy different positions within the causal and governance architecture, each requiring assessment according to the duties and legal rules applicable to that actor. Patient choices may remain relevant to shared decision-making or causal analysis in particular cases, but they should not be treated as a default transfer of professional or organisational responsibility. The objective should not be to solve this complexity by identifying a single human “last mile” onto whom the system transfers all accountability.

## 9. Preserving Human Agency: Critical–Reflective Thinking, Dialectic, and Teleology

### 9.1. Critical–Reflective Thinking

Critical–reflective thinking is proposed as a capacity to interrogate both the object of judgment and the judging subject. The critical question asks: Why should this conclusion be considered true? The reflective question asks: Why am I accepting this conclusion? This formulation overlaps with metacognition and reflective practice but gives special emphasis to preserving epistemic independence in human–AI systems.

For the present paper, this remains a research implication rather than a demonstrated safety mechanism. One empirical hypothesis is that structured critical–reflective interventions may reduce inappropriate human–AI convergence and correlated error. The stochastic model and clinical testing of this hypothesis belong to subsequent studies.

### 9.2. Dialectical Thinking in a Platonic Sense

Dialectical thinking is used here in a Platonic sense: inquiry that does not stop at appearances or isolated particulars but moves through distinctions toward intelligible structure and what is essential [51]. In high-tech medicine, such capacity is relevant because increasingly rich data can enlarge the accidental features available to attention while making it harder to recognise what is structurally decisive in the case.

The dialectical capacity therefore complements AI rather than competing with it. The system can expand the space of correlations; the physician must still discriminate what is essential for judgment.

### 9.3. Teleology and the Common Good

Technological governance ultimately requires an account of ends. Without teleology, optimisation becomes circular: the system optimises what has been selected because it has been selected. Medicine, by contrast, is a practice ordered toward goods internal to care and toward the good of the patient within a social and institutional common good.

The clinical good should not be imposed paternalistically by the physician nor reduced to an isolated preference. It is deliberated through the integration of evidence, the intrinsic goods of medicine, patient values and choices, proportionality, justice, and the concrete circumstances of care. This is the normative environment in which “best practice” becomes intelligible.

## 10. Implications for Healthcare

### 10.1. Clinical Practice

The physician’s professional centre of gravity may move progressively from producing all relevant inferences personally toward validating, integrating, contextualising, contesting, and responsibly acting on inferences produced within a hybrid system. This does not make clinical knowledge less important; it makes deep domain knowledge indispensable for supervision.

### 10.2. Medical Education

AI literacy is necessary but insufficient. Curricula should explicitly develop epistemic competence, normative deliberation, anthropological–relational understanding, systemic–hermeneutic interpretation, and critical–reflective thinking. The educational objective is not resistance to technology but the capacity to govern it.

### 10.3. Healthcare Organisations

Meaningful human oversight must be designed organisationally. Clinicians require time, authority, training, escalation pathways, audit mechanisms, and access to information sufficient to challenge automated outputs. A formally human-in-the-loop process may remain substantively machine-led if these conditions are absent.

### 10.4. Policy and Regulation

Responsibility should not be allocated simply by placing a human signature at the end of an algorithmic pathway. Regulation should consider the competence and conditions required for effective oversight, the distribution of responsibility across actors, and the jurisdiction-specific evolution of professional exigibility.

### 10.5. Innovation

The four competencies should not be treated as the residue left to humans after automation. Innovation itself may depend on the human capacity to identify that a problem has been framed incorrectly, formulate new ends, recognise ethically important discontinuities, and reinterpret context. A paradox is therefore possible: increasing artificial capability accompanied by declining human capacity to generate the conceptual discontinuities needed for further responsible innovation.

### 10.6. When Does the Four-Domain Framework Make a Difference?

The contribution of the four-domain framework becomes particularly salient when technological performance alone is insufficient to determine responsible medical judgment. Existing approaches to trustworthy AI, clinical evaluation, explainability, and human oversight remain essential. The proposed framework complements these approaches by examining whether the conditions required to translate technological outputs into responsible clinical action are adequately addressed.

For instance, a technically reliable and explainable prediction does not, in itself, establish whether acting upon it would be proportionate or beneficial for the patient (Truth and Good). Similarly, personalised algorithmic recommendations do not necessarily ensure recognition of the patient as an active participant in care (Person). While an output may be statistically valid, its clinical relevance also depends on whether the underlying data adequately represent the patient’s circumstances and whether the available evidence can be appropriately interpreted within the specific care setting (Context).

These distinctions also have organisational and medico-legal implications. Formal human oversight may be inadequate when clinicians lack the competence, information, time, or authority required to critically assess AI outputs. Furthermore, the existence of a validated AI system does not automatically establish a professional duty to use it. Professional exigibility therefore requires a separate assessment of what can reasonably be expected of clinicians under the applicable scientific, clinical, organisational, and legal circumstances.

The framework’s contribution lies in making explicit the additional questions that arise when technological outputs inform medical judgment and action, particularly where technical adequacy alone does not determine clinical appropriateness, meaningful human oversight, or professional obligations. These implications remain conceptual rather than empirically demonstrated comparative effects.

## 11. Conceptual Claims and Future Empirical Hypotheses

The framework is intended as a theoretical foundation for work still to be done. Conceptual claims and empirical hypotheses must therefore remain distinct.

### 11.1. Conceptual Claims

- Technological augmentation is normatively indeterminate: it can strengthen beneficial or harmful action.
- Functional similarity between AI interaction and human relational effects does not establish ontological equivalence between interaction and interpersonal relationship.
- Responsibility in sociotechnical medicine can be personal without being exclusive and distributed without being dissolved.
- Professional exigibility is dynamic and must be specified within national medico-legal systems as evidence, validated technologies, organisational conditions, and professional standards evolve.

### 11.2. Empirical Hypotheses for Subsequent Studies

- Human–AI systems with high error dependence will be less robust than systems with comparable component accuracy but greater epistemic independence.
- Structured critical–reflective interventions may reduce inappropriate human–AI convergence under defined conditions.
- Education focused on the four governance competencies may improve adaptive performance in technology-rich clinical environments compared with AI literacy alone.
- AI-mediated clinical workflows incorporating a cross-cutting governance layer structured around Truth, Good, Person, and Context may improve the identification of epistemic limitations, normative trade-offs, patient-specific considerations, and contextual constraints compared with otherwise equivalent AI-supported workflows without such a layer. This hypothesis could be tested through controlled clinical simulations and prospective studies assessing decision quality, critical–reflective reasoning, automation bias, and the effectiveness of human oversight.
- Organisations that preserve higher-order human judgment may identify novel problems and governance failures more effectively than organisations that maximise cognitive delegation without equivalent investment in human capability.

Structured stakeholder evaluation involving clinicians, patients and public representatives, healthcare organisations, medico-legal experts, regulators, and AI developers could assess the comprehensibility, completeness, practical relevance, and applicability of the four domains to clinical decision-making.

## 12. Limitations

This is a theoretical concept paper and does not establish causal effects. The technological horizon remains uncertain, especially regarding the speed and clinical relevance of embodied AI and quantum computing. The labour-market and industry sources discussed are deliberately selected complementary lenses rather than a systematic evidence synthesis, and they do not directly predict medical work. Consequently, their relevance to healthcare remains illustrative and cannot substitute for empirical evidence on changes in clinical roles and professional practice.

The personalist anthropology used here is an explicitly chosen philosophical framework and is not presented as metaphysically neutral. The relation–interaction distinction therefore depends on substantive claims about personhood and participation that remain open to philosophical disagreement.

Professional exigibility is proposed as an analytical medico-legal category, not as an already harmonised transnational doctrine. Its legal content must be developed within national systems. The comparative discussion of healthcare entitlements illustrates jurisdictional embeddedness but should not be conflated with professional duty. Moreover, the medico-legal discussion is selective and does not constitute a comprehensive doctrinal or jurisprudential analysis across jurisdictions.

Finally, the four-domain framework has not yet been empirically validated, nor has it undergone structured evaluation by relevant stakeholders; patients and public representatives were not directly involved in its development. The proposed role of critical–reflective thinking in preserving epistemic independence between clinicians and AI remains a hypothesis for subsequent mathematical simulation and clinical experimentation.

## 13. Conclusions

The ethical challenge of AI-driven medicine is not primarily to identify which human capacities technology cannot replace. It is to identify which human competencies become increasingly necessary to govern technological capability as that capability expands.

This paper proposes four: epistemic competence oriented to Truth; normative competence oriented to the Good; anthropological–relational competence oriented to the Person; and systemic–hermeneutic competence oriented to Context. Together, they provide a conceptual basis for responsible medical judgment in a system where inference is increasingly distributed across human and artificial agents, while their practical applicability and effectiveness remain to be empirically evaluated.

The physician should therefore not become the human terminal of an algorithmic process, and the patient should not become the passive recipient of an optimised pathway. The more powerful technology becomes, the more important it is that both professional and patient can take part—participate—in understanding, deliberation, and responsible action.

## Figures and Tables

**Table 1:** Selected approaches to AI in healthcare and their relationship to the proposed framework.

Approach	Primary Focus	Relationship to the Present Framework
WHO Ethics and Governance of AI for Health (2021) [2]	Ethical principles and institutional governance of healthcare AI.	Provides an ethical governance perspective relevant to responsible medical action.
FUTURE-AI (2025) [3]	Trustworthy development, evaluation, deployment, and monitoring of healthcare AI.	Addresses system trustworthiness and implementation requirements that complement the human competencies examined here.
DECIDE-AI (2022) [4]	Reporting early clinical evaluation of AI-based decision-support systems.	Addresses clinical utility, safety, and human factors in evaluating AI systems used in care.
Göndöcs and Dörfler (2024) [5]	Algorithmic prediction, diagnostic judgment, and professional responsibility.	Examines the distinction between prediction and judgment, which the present framework places within a broader analysis of medical action.
Proposed four-domain framework	Human governance of AI-mediated medical judgment and action.	Integrates Truth, Good, Person, and Context, linking responsible judgment, technological innovation, and professional exigibility.

**Table 2:** Four analytically irreducible questions and constitutive governance domains in AI-driven medicine.

Foundational Question	Governance Domain	Core Medical Meaning	Illustrative Applications
What is true?	Epistemic competence	Responsible judgment about evidence, uncertainty, validity, and limits.	Diagnosis; clinical-trial data; epidemiology; AI output validation; informed consent; follow-up.
What ought to be done?	Normative competence	Identification of the good and of right action within professional excellence.	Best practice; proportionality; therapeutic choice; resource use; safety; justice.
Who is the subject of care?	Anthropological–relational competence	Recognition of the patient as a person and participant, not a data object.	Humanisation; patient–physician relationship; autonomy; rights; shared decision-making.
What does this mean here and now?	Systemic–hermeneutic competence	Interpretation of correlations and evidence within a concrete, changing system and biography.	Comorbidity; family; organisation; culture; resources; patient history; follow-up.

## Data Availability

No new data were created or analyzed in this study. Data sharing is not applicable to this article.
